# Effectiveness of partial splenic embolization in colorectal cancer patients with chemotherapy-induced thrombocytopenia: results of a single institution retrospective study

**DOI:** 10.3389/fonc.2024.1468744

**Published:** 2024-10-28

**Authors:** Dan Li, Tao Peng, Ke-tong Wu, Yu-ting Huang, Yang Liu, Yuan Wan, Bo Zhang

**Affiliations:** ^1^ Department of General Surgery (Intervention Department), The Sixth Affiliated Hospital, Sun Yat-sen University, Guangzhou, China; ^2^ Biomedical Innovation Center, The Sixth Affiliated Hospital, Sun Yat-sen University, Guangzhou, China

**Keywords:** partial splenic embolization (PSE), chemotherapy-induced thrombocytopenia (CIT), colorectal cancer, platelet, cirrhosis

## Abstract

**Objective:**

To evaluate the safety and efficacy of partial splenic embolization (PSE) in treating chemotherapy-induced thrombocytopenia (CIT) in patients with colorectal cancer who failed to respond to platelet growth factor therapy.

**Methods:**

56 patients who underwent PSE were retrospectively analyzed. Based on the inclusion and the exclusion criteria, 29 patients were eligible for the study, of whom one underwent twice PSE procedures due to recurrent thrombocytopenia. The clinical characteristics were retrospectively analyzed with respect to efficacy, safety and outcome.

**Results:**

60.0% of patients restarted antineoplastic therapy after PSE. There was a positive correlation between difference value of platelet count and embolization material size (Eta Squared = 0.252, *p* < 0.05). The correlation between the absolute volume of spleen embolized and postoperative complications was analyzed, with a statistically significant result (*p* < 0.001). The mean preoperative spleen volume, the preoperative platelet count, postoperative platelet count and difference value of platelet count in the non-cirrhotic group were larger than those in the cirrhotic group (*p* < 0.001). The mean overall survival was 47.7 ± 7.7 months.

**Conclusion:**

PSE is safe and effective in the treatment of CIT patients with colorectal cancer. The larger the embolized particle, the more platelets grew. The severity of complication was also positively correlated with the absolute volume of spleen embolized. Therefore, large particle embolization materials can be used to improve the efficacy of PSE and reduce complications. For CIT patients with cirrhosis, PSE was less effective in improving platelet count than those without cirrhosis.

## Introduction

Chemotherapy-induced thrombocytopenia (CIT), a platelet counts of < 100×10^9^/L in peripheral blood, usually results from the inhibition of megakaryocytes after antitumor chemotherapy drugs (1). In recent years, the prevalence of CIT in patients with colorectal cancer was 61.7% in China. CIT increases bleeding complications and leads to delayed or reduced chemotherapy. Clinically, chemotherapy is often delayed or the doses reduced when the platelet count is between 75×10^9^/L and 100×10^9^/L, and most chemotherapy is stopped when the platelet count is < 50×10^9^/L (2). The mechanisms of chemotherapy drugs can affect all aspects of thrombocytopenia, including inhibiting the proliferation of hematopoietic stem and megakaryocyte progenitor cells, reducing the production of megakaryocytes, and inhibiting the production and release of platelets by megakaryocytes, ultimately leading to thrombocytopenia (3).

Administering the platelet growth factor can effectively improve the platelet count. However, this treatment is ineffective in some patients, with the platelet count remaining low or repeatedly decreasing, affecting the progress of chemotherapy. Splenectomy or partial splenic embolization (PSE) may be an effective therapy for such patients. Besides the extensive experience with PSE in patients with non-cancer hypersplenism, several reports about using PSE in oncology have been published. However, PSE still lacks data for CIT in patients with colorectal cancer (4–9).

The study retrospectively examined the efficacy and safety of PSE in treating CIT in patients with colorectal cancer who failed to respond to platelet growth factor therapy.

## Materials and methods

### Patients

**Figure 1:**
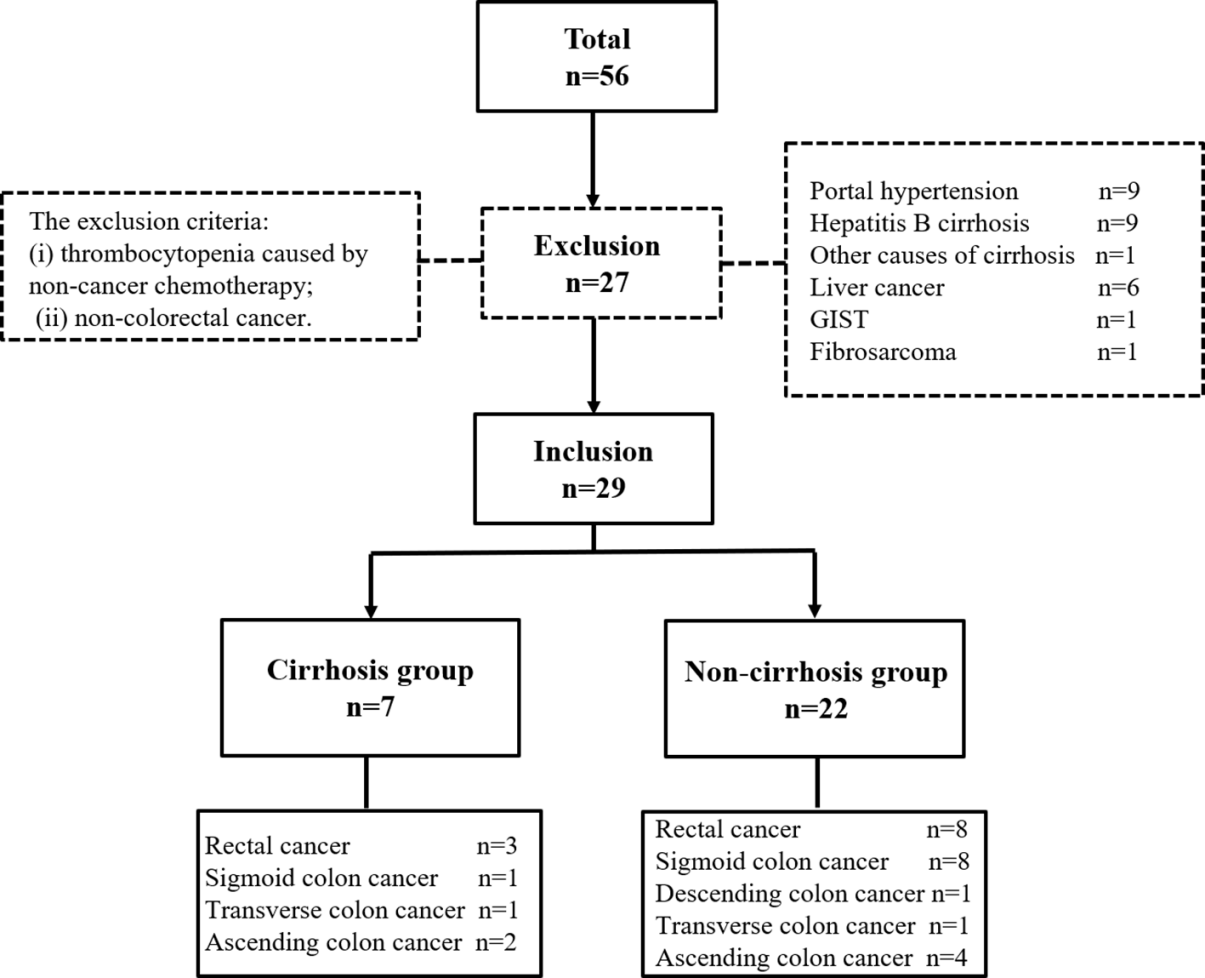
The inclusion and exclusion of patients to form the final cohort.

This study was approved by the Institutional Review Board and received a consent waiver for medical record review. The medical records of 56 patients who underwent PSE treatment from August 1, 2015 to January 31, 2024 at a single institution were retrospectively reviewed and analyzed. The inclusion criteria were (i) definite diagnosis of splenomegaly by imaging; (ii) abdominal computed tomography (CT) within one month before PSE; and (iii) more than three months of platelet growth factor therapy. The exclusion criteria were (i) thrombocytopenia caused by non-cancer chemotherapy; and (ii) non-colorectal cancer. Finally, 29 patients (19 male and 10 female) aged 56.9 ± 10.6 years (mean ± standard deviation) were treated in 30 PSE procedures. Because some patients with colorectal cancer had cirrhosis, the patients were classified into two groups based on whether they were complicated by liver cirrhosis: cirrhosis and non-cirrhosis. The non-cirrhosis group comprised patients with thrombocytopenia caused only by colorectal cancer chemotherapy. The cirrhosis group comprised patients with colorectal cancer and hepatitis cirrhosis with a baseline platelet count of > 100×10^9^/L before antineoplastic therapy (**Figure 1**). Cirrhosis was also diagnosed by imaging and was not confirmed by biopsy.

### Preoperative spleen measures

Splenomegaly was divided into three grades based on the maximum spleen length measured on the preoperative CT in the axial view (10): mild (12 – 13 cm), moderate (13 – 15 cm), and severe (> 15 cm). In addition, preoperative splenic volumes were calculated using a previously described formula (11): splenic volume (in cm^3^) = 30 + 0.58 (W × L × Th). The preoperative splenic volume was manually measured by two radiologists with three years of experience reading abdominal CT scans using the Picture Archiving and Communication System, and the reproducibility and consistency of their measurements were demonstrated.

### PSE procedure

All patients underwent PSE using a single bottle of gelatin sponge (Gelfoam), or polyvinyl alcohol (PVA), or microspheres (CalliSpheres) (12). Two interventional radiologists with eight years of experience performed PSE by selecting one of the three materials according to their personal preference. After obtaining written informed consent from 29 patients, selective splenic artery cannulation was performed via the femoral artery using the Seldinger technique. Then, splenic arteriography was used to assess the anatomy of the splenic artery. Finally, super-selective embolization was performed according to the arteriography results.

The intraoperative embolization proportion was divided into three grades based on the surgical records: small (an embolization volume of 30% – 40%, generally not < 30%), medium (an embolization volume of 40% – 60%), and large (an embolization volume of > 60%). The absolute volume of spleen embolized is calculated as the pre-PSE splenic volume multiplied by the percentage of the spleen that was embolized. In addition, the embolization material specification (size) was also divided into three grades: small (≤ 500μm), medium (500 – 700μm), or large (> 700μm) for gelatin sponge and microspheres. If the embolization material was PVA, size was small (≤ 560μm), medium (560 – 710μm), or large (> 710μm).

### Follow-up and evaluations

Whether treatment could be resumed and its interval were followed up. The study cutoff date was January 2024. The follow-up was based on the examination and treatment records of patients returning to the hospital. Patients who could not return to the hospital were followed up by telephone.

### Outcome measures

This study’s primary objective was to determine whether patients with colorectal cancer benefited from PSE. The efficacy and safety of PSE were evaluated by comparing platelet counts before and after PSE, whether to receive treatment again after PSE and the time, and PSE complications. Technical success was defined as successful super-selective cannulation of the splenic artery branch. Clinical success was defined as an increase in platelet count to 100×10^9^/L (13), or the ability to be retreated. Therefore, patients who continued anti-tumor therapy after PSE were classified into the restart treatment group, otherwise they were classified into the non-restart treatment group. Clinical failure was defined as not meeting the criteria for clinical success.

This study’s secondary outcomes were whether preoperative platelet count and recovery after PSE differed between the cirrhosis and non-cirrhosis groups.

### Statistical analysis

Statistical analyses were performed using SPSS for Windows software (version 22.0; IBM, Armonk, NY, USA). All quantitative data are reported as the mean ± standard deviation. All qualitative data are reported as the frequency or percentage. Normally distributed continuous variables with homogeneous variance were compared between groups using the *t*-test. Continuous variables with a non-normal distribution or nonhomogeneous variance were compared between groups using the Mann–Whitney test. Pearson’s correlation coefficient was used to assess correlations for quantitative data, and Eta nonparametric correlation coefficient was used to assess correlations for qualitative data. Kaplan–Meier curves were used to determine the overall survival probability and median survival. A *P*-value of <0.05 was considered statistically significant.

## Results

### Primary outcomes analysis

#### Patient demographics

Twenty-nine patients were treated with 30 PSE procedures; one underwent two PSE procedures due to recurrent thrombocytopenia. The indication for PSE in these patients was that they were unable to continue antineoplastic therapy because of thrombocytopenia and were unresponsive to conservative treatment for at least 3 months. The technical success rate was 100%, and the clinical success rate was 90%. 96.7% (29 of 30) patients presented elevation in the number of platelets after PSE. 60.0% (18 of 30) patients had received further cancer treatments. The mean time between PSE and retreatment was 42.9 ± 34.1 days. Demographics in the study were summarized in **Table 1**.

**Table 1:** Demographics and clinical features of the patients.

Variables	Values
**Age (years)**	56.9 ± 10.6
**Sex (male/female)**	19/10
**BMI**	23.5 ± 2.8
**Primary**	29
Rectal cancer	11
Sigmoid colon cancer	9
Descending colon cancer	1
Transverse colon cancer	2
Ascending colon cancer	6
**Spleen volume (cm^3^)**	696.9 ± 224.7
**Degree of hypersplenism (maximum spleen length)** ^*^	
Mild	3
Moderate	13
Severe	14
**Embolization proportion (small/medium/large)**	12/13/5
**Preoperative platelet count (×10^9^/L)**	60.1 ± 21.5
**Postoperative platelet count (×10^9^/L)**	135.1 ± 63.1
**Difference value of platelet count (×10^9^/L)**	75.0 ± 64.2
**Days of platelet count recovery (days)**	5.0 ± 3.6
**Time between PSE and retreatment (days)**	42.9 ± 34.1
**Complications**	
Abdominal pain	23
Constipation and abdominal distension	10
Fever	10

^*^Twenty-nine patients were treated with 30 PSE procedures; one underwent two PSE procedures due to recurrent thrombocytopenia.

Two radiologists with three years of experience measured the maximum length and volume of the spleen, and the correlation of their measurements is shown in **Supplementary Figures S1**, **S2**. The intra-class correlation coefficient (ICC) was used to evaluate the consistency between the two observers. The ICCs for maximum spleen length and preoperative splenic volume were 0.918 (*p* < 0.001) and 0.893 (*p* < 0.001), respectively. Therefore, the consistency of the two radiologists’ measurements of the maximum length and volume of the spleen was high (i.e., the stability of the measurement data between the two observers was good). Therefore, the two data sets were meaned for subsequent analyses.

The mean preoperative spleen volume was 696.9 ± 224.7 cm^3^. According to the classification of maximum spleen length, there was 3 cases of mild splenomegaly, 13 cases of moderate splenomegaly, and 14 cases of severe splenomegaly. The mean post-procedural platelet count (135.1 ± 63.1×10^9^/L) was twice that one week before PSE (60.1 ± 21.5×10^9^/L). The mean time of platelet count recovery after PSE was 5.0 ± 3.6 days.

**Figure 2:**
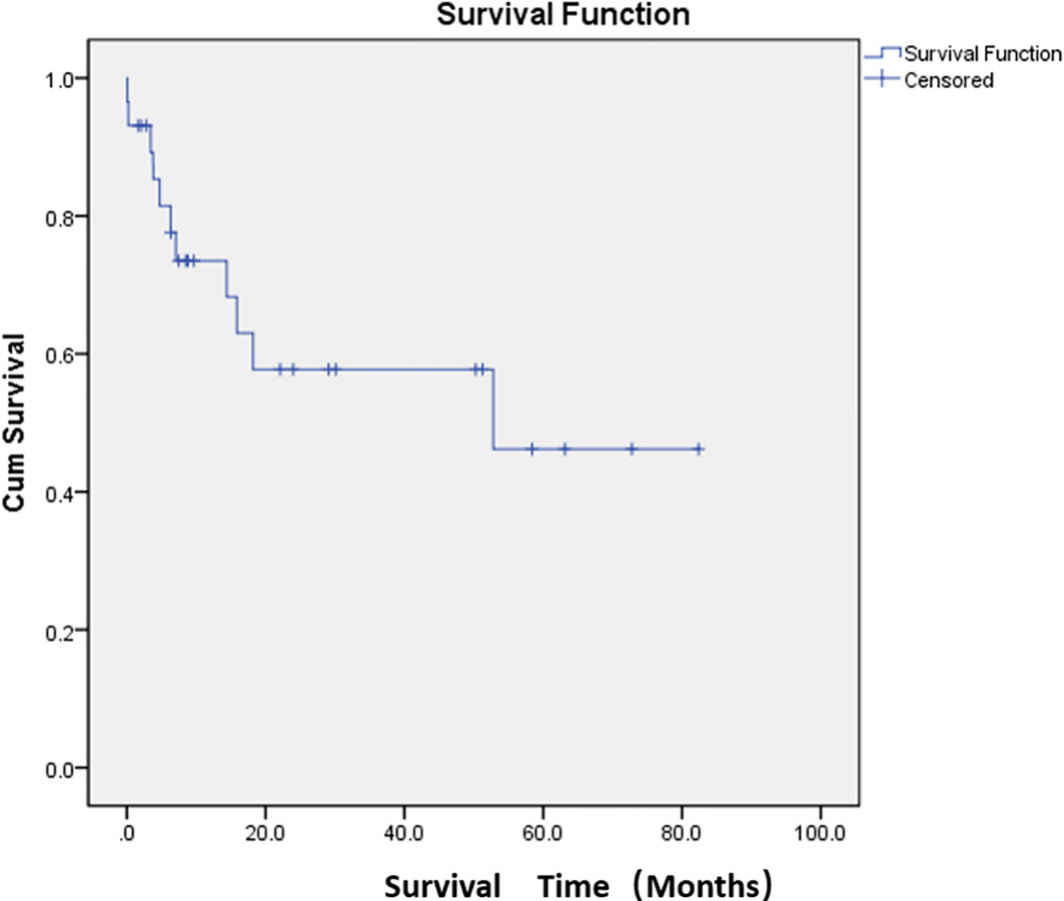
Median overall survival after embolization. Kaplan–Meier curve demonstrating overall survival probability for the study population.

**Figure 3:**
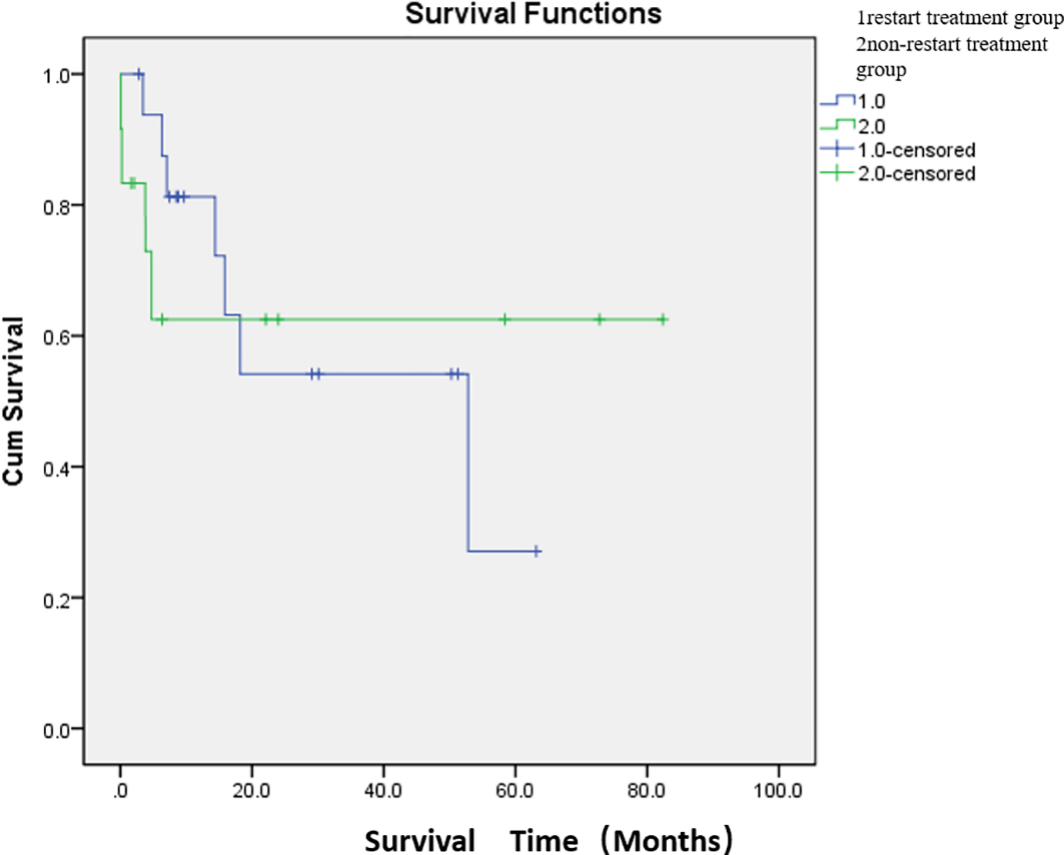
Comparison of median survival between the two groups after embolization. There were 17 patients in the restart treatment group (one patient underwent two PSE) and 12 patients in the non-restart treatment group. The median survival time was 36.8 months in the restart treatment group and 52.4 months in the non-restart treatment group. There was no significant difference in survival between the restart treatment and non- restart treatment groups (*p* > 0.05).

Adverse events were defined as postoperative complications related to PSE. The most common complication was abdominal pain (76.7%). The other complications were constipation and abdominal distension (33.3%) and fever (33.3%). The cutoff date was defined as death date due to cancer or January 2024. Eleven patients died of cancer before the end of the study. The median overall survival was 47.7 months (**Figure 2**; **Supplementary Table S1**). The median survival time was 36.8 months in the restart treatment group and 52.4 months in the non-restart treatment group (**Figure 3**; **Supplementary Table S2**), and there was no significant difference (*p* > 0.05).

#### Effect of embolization volume and material on platelet count and postoperative complications

Preoperative spleen volume, embolization volume and embolization material may have an impact on the difference value of platelet count. Pearson correlation analysis was used to analyze between preoperative spleen volume and the difference value of platelet count, the absolute volume of spleen embolized and the difference value of platelet count, and the r value was 0.311(*p* = 0.05) and 0.027 (*p* = 0.445), respectively. In addition, Pearson analysis was used to analyze the correlation between preoperative spleen volume and platelet count, and the r value was 0.006(*p* = 0.488) (**Table 2**). Thus, there may be a weak positive correlation between preoperative spleen volume and the difference value of platelet count. However, there was no significant correlation between absolute volume of spleen embolized and the difference value of platelet count, and preoperative spleen volume and platelet count.

**Table 2:** Effect of preoperative spleen volume and absolute volume of spleen embolized on difference value of platelet count.

	Mean ± SD	r	P
Preoperative spleen volume (cm^3^)	705.8 ± 223.1	0.311	0.050
Difference value of platelet count (×10^9^/L)	78.4 ± 62.5		
Absolute volume of spleen embolized (cm^3^)	338.7 ± 125.9	0.027	0.445
Difference value of platelet count (×10^9^/L)	78.4 ± 62.5		
Preoperative spleen volume (cm^3^)	696.9 ± 224.7	0.006	0.488
Preoperative platelet count (×10^9^/L)	60.1 ± 21.5		

The effect of embolization proportion, material type and material size on the difference value of platelet count were analyzed by correlation ratio. The difference value of platelet count was 64.1 ± 29.3×10^9^/L with small embolic particles, 80.7 ± 84.3×10^9^/L with medium embolic particles, and 188.0 ± 65.1×10^9^/L with large embolic particles. The difference value of platelet count was significantly positively correlated with embolization material size (Eta Squared = 0.252, *p* < 0.05). Therefore, the larger the embolic particles, the greater the difference in platelet count (i.e., the better the platelet count recovery). However, embolization proportion and material type did not significantly affect the difference in the difference value of platelet count (*p* > 0.05) (**Table 3**).

**Table 3:** Effect of embolization proportion and embolic material on difference value of platelet count.

	Grade (n)	Mean ± SD	Eta Squared	P
Embolization proportion	small (12)	92.0 ± 54.8	0.040	0.587
	medium (12)	72.6 ± 76.5		
	large (5)	59.6 ± 43.6		
Embolic material size	small (17)	64.1 ± 29.3	0.252	0.023
	medium (10)	80.7 ± 84.3		
	large (2)	188.0 ± 65.1		
Embolic material type	gelatin sponge (1)	142.00	0.038	0.601
	PVA (17)	75.6 ± 63.1		
	microsphere (11)	76.8 ± 64.4		

Chi-square test was used in the analysis of factors influencing postoperative complications (including abdominal pain, constipation and abdominal distension, and fever), and the results showed that P values were greater than 0.05. The embolization proportion and material did not significantly affect the occurrence of postoperative complications (**Table 4**). The effect of the absolute volume of spleen embolized on the postoperative complications was analyzed by correlation ratio, and P values were less than 0.001. The absolute volume of spleen embolized had the strongest correlation with the occurrence of pain, with a positive correlation (**Table 5**).

**Table 4:** Effect of embolization proportion and embolic material on postoperative complications.

	Complications	P value
Embolization proportion	Abdominal pain	0.188
	Constipation and abdominal distension	0.580
	Fever	0.758
Embolic material size	Abdominal pain	0.207
	Constipation and abdominal distension	0.522
	Fever	0.058
Embolic material type	Abdominal pain	0.175
	Constipation and abdominal distension	0.761
	Fever	0.268

**Table 5:** Effect of absolute volume of spleen embolized on postoperative complications.

	Grade	Mean ± SD	Eta Squared	P
Abdominal pain^*^	0	253.0 ± 33.3	0.248	< 0.001
	1	375.1 ± 109.4		
	2	394.5 ± 130.2		
	3	473.7 ± 120.9		
Constipation and abdominal distension^#^	0	358.9 ± 133.0	0.046	< 0.001
	1	417.3 ± 122.7		
Fever^#^	0	395.0 ± 129.1	0.027	< 0.001
	1	348.2 ± 133.8		

^*^Pain was graded into four levels based on whether pain medication was needed after surgery and the level of pain medication: 0 for no pain, 1 for mild pain, 2 for moderate pain, and 3 for severe pain.

^#^Fever, constipation and abdominal distention were classified into two categories: 0 represented absence and 1 represented occurrence.

### Secondary outcomes analysis

The t test or rank sum test (Mann–Whitney test) was used to compare the cirrhosis group with the non-cirrhosis group. The distributions of sex, degree of hypersplenism, embolization proportion and days of platelet count recovery did not differ significantly between the cirrhosis and non-cirrhosis groups (*p* > 0.05). The age and BMI of the non-cirrhotic group were larger than those of the cirrhotic group, and the differences were statistically significant (*p* < 0.05) (**Table 6**).

**Table 6:** Comparisons between the two subgroups (cirrhosis group and non-cirrhosis group).

Variables	Cirrhosis group	Non-cirrhosis group	P
Age (years)	58.0 ± 10.0	61.4 ± 7.8	<0.001
Sex (male/female)	6/1	13/9	0.367
BMI	23.6 ± 2.4	24.5 ± 3.3	<0.001
Spleen volume (cm^3^)	732.9 ± 186.2	873.0 ± 388.5	<0.001
Degree of hypersplenism ^a^	0/5/2	2/11/10	0.480
Embolization proportion ^b^	3/2/2	9/11/3	0.534
Preoperative platelet count (×10^9^/L)	62.6 ± 11.8	81.1 ± 30.0	<0.001
Postoperative platelet count (×10^9^/L)	157.9 ± 75.6	180.0 ± 79.9	<0.001
Difference value of platelet count	121.4 ± 83.7	141.4 ± 78.1	<0.001
Days of platelet count recovery (days)	7.4 ± 5.1	7.4 ± 2.8	0.228

^a^“Degree of hypersplenis” was classified as mild, moderate, and severe.

^b^“Embolization proportion” was divided into small, medium, and large.

The mean preoperative spleen volume was significantly larger in the non-cirrhosis group (873.0 ± 388.5 cm^3^) than in the cirrhosis group (732.9 ± 186.2 cm^3^; *p* < 0.001). In addition, the preoperative platelet count, postoperative platelet count and difference value of platelet count in the non-cirrhotic group were larger than those in the cirrhotic group, and the differences were statistically significant. (*p* < 0.001) (**Table 6**).

**Figure 4:**
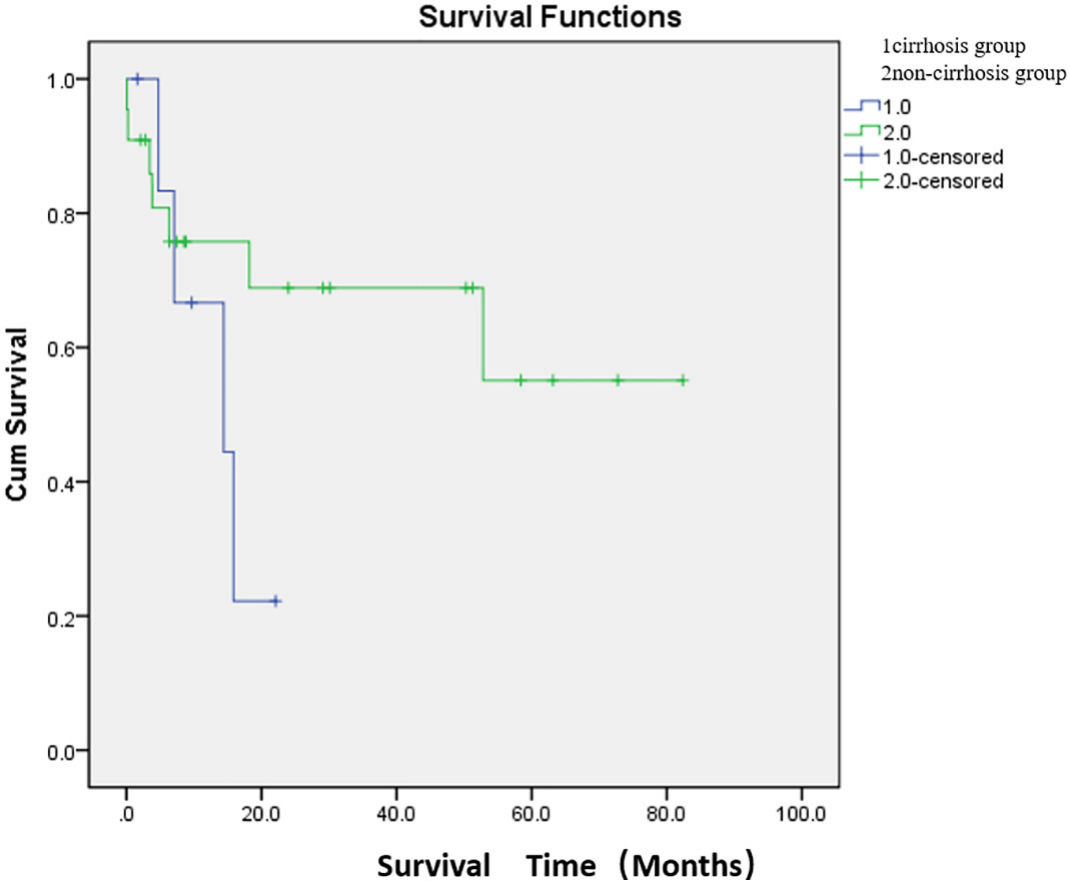
Comparison of median survival between the two groups after embolization. There were 7 patients in the cirrhosis group and 22 patients in the non-cirrhosis group (one patient underwent two PSE). The median survival time was 13.6 months in the cirrhosis group and 54.6 months in the non-cirrhosis group. There was no significant difference in survival between the cirrhosis and non-cirrhosis groups (*p* > 0.05).

The median survival time was 13.6 months in the cirrhosis group and 54.6 months in the non-cirrhosis group. There was no significant difference in survival between the cirrhosis and non-cirrhosis groups (*p* > 0.05) (**Figure 4**; **Supplementary Table S3**).

## Discussion

This study showed a clinical success rate of 90.0%, and the platelet count increased by twice times within a mean of five days after PSE. These findings were similar to previous reports in which the platelet count increased three times within one week (6, 7, 9, 13). In addition, more than half of the patients (60.0%) could continue to restart treatment, which was lower than in a previous study that 97.0% of patients restarted chemotherapy and 3.0% improved preoperative platelet counts (14). Among the 12 patients in this study who did not restart treatment, 10 patients gave up continuing antineoplastic therapy because of financial reasons or concerns about complications, and 2 patients died of cancer. In the study, patients in the non-restart treatment group had a higher survival rate than those in the restart treatment group. It may be the small sample size and the presence of maxima in the non-restart treatment group (**Supplementary Material**). In addition, there was no statistically significant difference between the two groups to assess the effect of subsequent restarting therapy on survival. Three patients in this study were clinical failures. A previous study found in which lower preoperative platelet values and splenic tissue embolism rates were risk factors for treatment failure (14). In these three cases, the lowest platelet count was 6.0×10^9^/L.

The post-embolization syndrome is mainly due to splenic necrosis and inflammatory effusion. The primary complications after PSE were abdominal pain, constipation and abdominal distension, and fever. However, most patients could tolerate them without additional therapy, or their symptoms were significantly relieved after drug treatment. Among them, abdominal pain had the highest incidence, possibly due to the complete infarction of the embolized organ caused by embolization (12). However, there were no significant differences in the incidences of abdominal pain (*p* = 0.175), constipation and abdominal distension (*p* = 0.761), and fever (*p* = 0.268) among the three embolization materials. A previous study reported that permanent particles were associated with greater abdominal pain, but fevers were more severe with gelatin sponges than with permanent particles (12). There were some differences between the results of this study and the previous results, which may be related to the unequal number of cases among the groups in this study, and only one patient used gelatin sponge. In addition, the study found that although the embolization proportion had no significant effect on postoperative complications, the absolute volume of spleen embolized had a statistically significant difference on postoperative complications (*p* < 0.001). For patients without pain in this study, the mean absolute volume of spleen embolized was 253.0 ± 33.3 cm^3^; for mild pain, the absolute volume of spleen embolized was 375.1 ± 109.4 cm^3^; for moderate pain, the absolute volume of spleen embolized was 394.5 ± 130.2 cm^3^; for severe pain, the absolute volume of spleen embolized was 473.7 ± 120.9 cm^3^. The larger the absolute volume of spleen embolized, the more obvious the abdominal pain and the more prone to constipation and abdominal distension.

Previous studies have reported a positive linear correlation between the increase in platelet count after PSE and splenic embolism volume (*r* = 0.3, *p* = 0.006) (14). The recommended spleen volume in PSE is about 50% – 70% (14–18). Thrombocytopenia may recur after PSE if < 50% of the spleen is embolized. Some studies have adopted a more cautious strategy, initially embolizing 30% – 50% of the spleen volume (19) and, if recurrent, increasing the embolizing volume to 70%. However, this study showed no significant association between embolization volume and difference value of platelet count, either in absolute volume of spleen embolized (*p* = 0.445) or in embolization proportion (*p* = 0.587). However, there may be a certain correlation between preoperative spleen volume and difference value of platelet count (r = 0.311, *p* = 0.050), but there was no significant correlation between preoperative spleen volume and preoperative platelet count (r = 0.006, *p* = 0.488). This can be explained by a larger spleen, indicating that thrombocytopenia is mainly due to increased destruction, while the function of platelet production is less affected. Therefore, the spleen’s destructive capacity is reduced, and the platelet count naturally increases significantly.

This study found a strong correlation between embolic material size and the difference value of platelet count (Eta Squared = 0.252, *p* = 0.023). Most previous studies have used embolic particles between 300 and 700μm. This study found a positive correlation between postoperative platelet recovery and embolization material size. Embolic particles of 300–500μm were chosen because the embolization target was the terminal artery (14). However, in patients undergoing PSE, the primary purpose is to reduce the spleen parenchyma and further reduce the destruction of platelets, thus increasing the platelet count, rather than dense embolization to cause “devascularization.” In addition, an excessive increase in splenic embolization volume may lead to a significant increase in platelet count, which may also increase the incidence of post-embolization complications. Therefore, a larger size of embolic materials instead of increasing the embolization volume, which can increase the platelet count and reduce postoperative complications.

Previous studies have reported reduced thrombopoietin levels in patients with cirrhosis, which affects thrombopoiesis (20). Therefore, thrombocytopenia may be further exacerbated by reduced thrombopoietin production in patients with CIT who also have underlying liver dysfunction. In this study, preoperative spleen volumes, preoperative platelet count, postoperative platelet count and difference value of platelet count differed significantly between the cirrhosis and non-cirrhosis groups (*p* < 0.001). The mean values of the above indexes in the non-cirrhotic group were higher than those in the cirrhotic group. The results further indicated that CIT patients with cirrhosis not only had increased platelet destruction but also decreased platelet production. Therefore, under the dual effect, even if the degree of splenomegaly is not as good as that of the non-cirrhotic group, the mean values of preoperative platelet count, postoperative platelet count and difference value of platelet count were smaller than that of the non-cirrhotic group. In addition, the mean survival time was longer in the non-cirrhotic group (54.6 months) than in the cirrhotic group (13.6 months), although there was no significant difference in the median survival time between the two groups.

This study’s main limitation was its retrospective design, which is subject to potential uncontrolled bias. In addition, this was a single-center study, and patient selection was at the clinical oncologist’s discretion. Finally, this study’s sample size was relatively small, both overall and in each group, and the number of patients in each group was not matched. Further randomized controlled studies with large samples are needed.

PSE effectively improved platelet count in CIT patients with colorectal cancer who failed to respond to platelet growth factor therapy. Antineoplastic therapy could be restarted in more than half of patients. There was a positive correlation between difference value of platelet count and embolization material size. The larger the embolized particle, the more platelets grew. The severity of complications was also positively correlated with the absolute volume of spleen embolized. However, most patients could tolerate them, or their symptoms were significantly relieved after drug treatment. Therefore, large particle embolization materials can be used to improve the efficacy of PSE and reduce complications. For CIT patients with cirrhosis, PSE was less effective in improving platelet count than those without cirrhosis due to its dual effects.

## Data Availability

The raw data supporting the conclusions of this article will be made available by the authors, without undue reservation.
