# Supplementary material for: Effectiveness of partial splenic embolization in colorectal cancer patients with chemotherapy-induced thrombocytopenia: results of a single institution retrospective study
Source: Front Oncol. 2024 Oct 28;14:1468744. doi: 10.3389/fonc.2024.1468744 (PMC11551041; doi:10.3389/fonc.2024.1468744)
Supplement: Supplementary file 1 [file DataSheet1.docx]

**FigureS1**


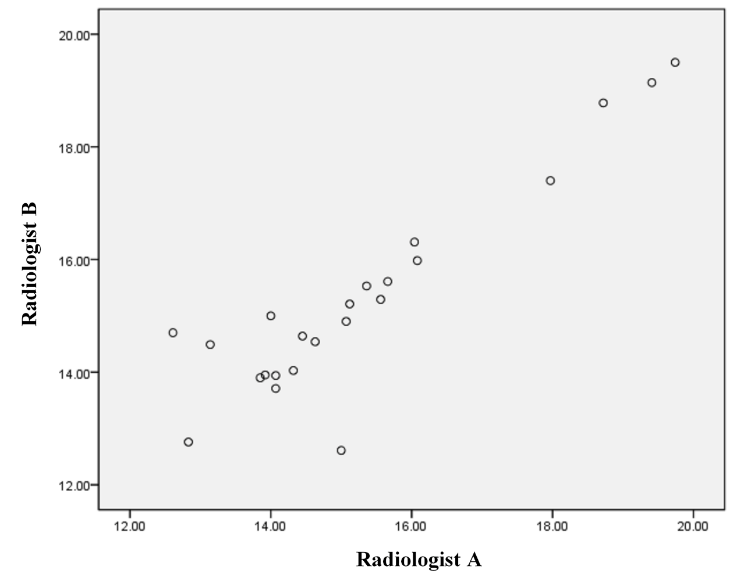


**FigureS2**


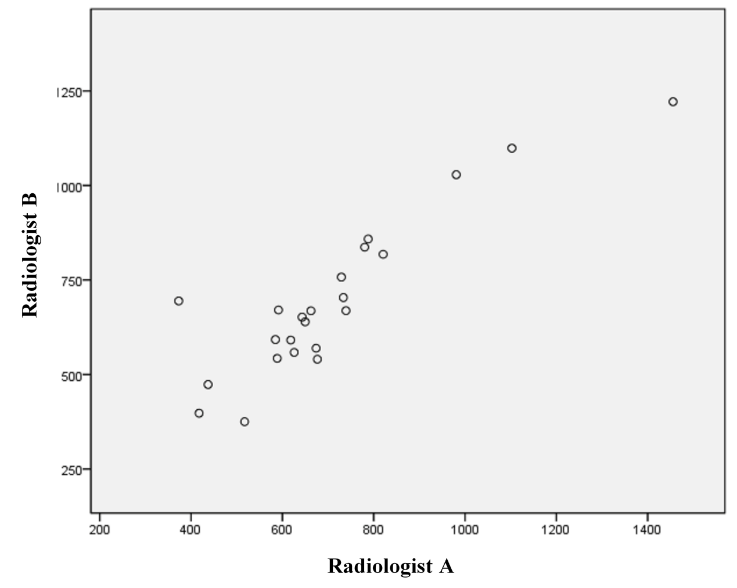


**Table S1**

| **Survival Table** | | | | | | |
| --- | --- | --- | --- | --- | --- | --- |
|  | Time | Status | Cumulative Proportion Surviving at the time | | N of Cumulative Events | N of Remaining  Cases |
|  |  |  | Estimate | Std. Error |  |  |
| 1 | .100 | 1 | .966 | .034 | 1 | 28 |
| 2 | .233 | 1 | .931 | .047 | 2 | 27 |
| 3 | 1.633 | 0 | . | . | 2 | 26 |
| 4 | 2.100 | 0 | . | . | 2 | 25 |
| 5 | 2.833 | 0 | . | . | 2 | 24 |
| 6 | 3.433 | 1 | .892 | .059 | 3 | 23 |
| 7 | 3.800 | 1 | .853 | .068 | 4 | 22 |
| 8 | 4.733 | 1 | .815 | .075 | 5 | 21 |
| 9 | 6.300 | 1 | .776 | .081 | 6 | 20 |
| 10 | 6.333 | 0 | . | . | 6 | 19 |
| 11 | 7.067 | 1 | .735 | .086 | 7 | 18 |
| 12 | 7.467 | 0 | . | . | 7 | 17 |
| 13 | 8.467 | 0 | . | . | 7 | 16 |
| 14 | 8.800 | 0 | . | . | 7 | 15 |
| 15 | 9.667 | 0 | . | . | 7 | 14 |
| 16 | 14.400 | 1 | .683 | .095 | 8 | 13 |
| 17 | 15.867 | 1 | .630 | .101 | 9 | 12 |
| 18 | 18.200 | 1 | .578 | .105 | 10 | 11 |
| 19 | 22.100 | 0 | . | . | 10 | 10 |
| 20 | 23.967 | 0 | . | . | 10 | 9 |
| 21 | 29.067 | 0 | . | . | 10 | 8 |
| 22 | 30.100 | 0 | . | . | 10 | 7 |
| 23 | 50.267 | 0 | . | . | 10 | 6 |
| 24 | 51.267 | 0 | . | . | 10 | 5 |
| 25 | 52.800 | 1 | .462 | .133 | 11 | 4 |
| 26 | 58.367 | 0 | . | . | 11 | 3 |
| 27 | 63.133 | 0 | . | . | 11 | 2 |
| 28 | 72.767 | 0 | . | . | 11 | 1 |
| 29 | 82.367 | 0 | . | . | 11 | 0 |

**Table S2**

| **Survival Table** | | | | | | | |
| --- | --- | --- | --- | --- | --- | --- | --- |
|  | | Time | Status | Cumulative Proportion Surviving at the Time | | N of Cumulative Events | N of Remaining Cases |
|  |  |  |  | Estimate | Std. Error |  |  |
| 1.0 | 1 | 2.833 | .0 | . | . | 0 | 16 |
|  | 2 | 3.433 | 1.0 | .938 | .061 | 1 | 15 |
|  | 3 | 6.300 | 1.0 | .875 | .083 | 2 | 14 |
|  | 4 | 7.067 | 1.0 | .813 | .098 | 3 | 13 |
|  | 5 | 7.467 | .0 | . | . | 3 | 12 |
|  | 6 | 8.467 | .0 | . | . | 3 | 11 |
|  | 7 | 8.800 | .0 | . | . | 3 | 10 |
|  | 8 | 9.667 | .0 | . | . | 3 | 9 |
|  | 9 | 14.400 | 1.0 | .722 | .122 | 4 | 8 |
|  | 10 | 15.867 | 1.0 | .632 | .136 | 5 | 7 |
|  | 11 | 18.200 | 1.0 | .542 | .143 | 6 | 6 |
|  | 12 | 29.067 | .0 | . | . | 6 | 5 |
|  | 13 | 30.100 | .0 | . | . | 6 | 4 |
|  | 14 | 50.267 | .0 | . | . | 6 | 3 |
|  | 15 | 51.267 | .0 | . | . | 6 | 2 |
|  | 16 | 52.800 | 1.0 | .271 | .204 | 7 | 1 |
|  | 17 | 63.133 | .0 | . | . | 7 | 0 |
| 2.0 | 1 | .100 | 1.0 | .917 | .080 | 1 | 11 |
|  | 2 | .233 | 1.0 | .833 | .108 | 2 | 10 |
|  | 3 | 1.633 | .0 | . | . | 2 | 9 |
|  | 4 | 2.100 | .0 | . | . | 2 | 8 |
|  | 5 | 3.800 | 1.0 | .729 | .135 | 3 | 7 |
|  | 6 | 4.733 | 1.0 | .625 | .151 | 4 | 6 |
|  | 7 | 6.333 | .0 | . | . | 4 | 5 |
|  | 8 | 22.100 | .0 | . | . | 4 | 4 |
|  | 9 | 23.967 | .0 | . | . | 4 | 3 |
|  | 10 | 58.367 | .0 | . | . | 4 | 2 |
|  | 11 | 72.767 | .0 | . | . | 4 | 1 |
|  | 12 | 82.367 | .0 | . | . | 4 | 0 |

1 represents the restart treatment group and 2 represents the non-restart treatment group.

**Table S3**

| **Survival Table** | | | | | | | |
| --- | --- | --- | --- | --- | --- | --- | --- |
|  | | Time | Status | Cumulative Proportion Surviving at the Time | | N of Cumulative Events | N of Remaining Cases |
|  |  |  |  | Estimate | Std. Error |  |  |
| 1.0 | 1 | 1.633 | .0 | . | . | 0 | 6 |
|  | 2 | 4.733 | 1.0 | .833 | .152 | 1 | 5 |
|  | 3 | 7.067 | 1.0 | .667 | .192 | 2 | 4 |
|  | 4 | 9.667 | .0 | . | . | 2 | 3 |
|  | 5 | 14.400 | 1.0 | .444 | .222 | 3 | 2 |
|  | 6 | 15.867 | 1.0 | .222 | .192 | 4 | 1 |
|  | 7 | 22.100 | .0 | . | . | 4 | 0 |
| 2.0 | 1 | .100 | 1.0 | .955 | .044 | 1 | 21 |
|  | 2 | .233 | 1.0 | .909 | .061 | 2 | 20 |
|  | 3 | 2.100 | .0 | . | . | 2 | 19 |
|  | 4 | 2.833 | .0 | . | . | 2 | 18 |
|  | 5 | 3.433 | 1.0 | .859 | .076 | 3 | 17 |
|  | 6 | 3.800 | 1.0 | .808 | .087 | 4 | 16 |
|  | 7 | 6.300 | 1.0 | .758 | .095 | 5 | 15 |
|  | 8 | 6.333 | .0 | . | . | 5 | 14 |
|  | 9 | 7.467 | .0 | . | . | 5 | 13 |
|  | 10 | 8.467 | .0 | . | . | 5 | 12 |
|  | 11 | 8.800 | .0 | . | . | 5 | 11 |
|  | 12 | 18.200 | 1.0 | .689 | .108 | 6 | 10 |
|  | 13 | 23.967 | .0 | . | . | 6 | 9 |
|  | 14 | 29.067 | .0 | . | . | 6 | 8 |
|  | 15 | 30.100 | .0 | . | . | 6 | 7 |
|  | 16 | 50.267 | .0 | . | . | 6 | 6 |
|  | 17 | 51.267 | .0 | . | . | 6 | 5 |
|  | 18 | 52.800 | 1.0 | .551 | .151 | 7 | 4 |
|  | 19 | 58.367 | .0 | . | . | 7 | 3 |
|  | 20 | 63.133 | .0 | . | . | 7 | 2 |
|  | 21 | 72.767 | .0 | . | . | 7 | 1 |
|  | 22 | 82.367 | .0 | . | . | 7 | 0 |

1 represents the cirrhosis group and 2 represents the non-cirrhosis group.
